# Thoracotomy for Urgent Repair of Lung Torsion Identified as an Incidental Finding

**DOI:** 10.7759/cureus.115787

**Published:** 2026-09-04

**Authors:** Armando Bunjaj, Nathan Booth, Shyamal Pansuriya, Corbin Cleary

**Affiliations:** 1 Department of Medical Education, Lake Erie College of Osteopathic Medicine, Elmira, USA; 2 Department of Surgery, Henry Ford Macomb Hospital, Clinton Township, USA; 3 Department of Surgery, Artesia General Hospital, Artesia, USA

**Keywords:** bronchoscopy, bulla, bullous disease, detorsion, lung torsion, thoracotomy

## Abstract

We report a 68-year-old male with chronic obstructive pulmonary disease on home oxygen who presented with foot swelling and erythema, associated with a new right foot wound. He denied chest pain and overt shortness of breath at the time of his presentation. Initial chest radiography obtained during the evaluation raised concern for pneumothorax versus a large bulla. Computed tomography with intravenous contrast and computed tomography angiography revealed an expanding right lower lobe bulla extending into the upper lung zone, narrowing of the right bronchial tree, and abnormal right hilar vasculature orientation concerning for lung torsion. Bronchoscopy demonstrated compression of the right upper lobe bronchus and severe compression of the right middle and lower lobe bronchi. The right lower and middle lobes were detorsed, and the bullous right lower lobe tissue was removed by wedge resection. Test inflation demonstrated satisfactory expansion, and there were no intraoperative complications. The postoperative course was complicated by a pleural effusion requiring decortication two weeks postoperatively, after which the patient recovered well. This case highlights how lung torsion may be identified incidentally and require urgent operative repair despite an inconspicuous respiratory presentation.

## Introduction

Lung torsion is an uncommon but potentially fatal entity in which a lobe, or rarely the entire lung, rotates around its bronchovascular pedicle, leading to airway obstruction and impairment of venous/arterial flow, with risk of hemorrhagic infarction and necrosis [1]. It is most often described after major disruption of the thoracic cavity (e.g., thoracotomy, lung transplantation, and significant trauma), but spontaneous torsion can occur in the setting of pneumothorax, pleural effusion, and lobar atelectasis [1]. The reported incidence of lung torsion is approximately 0.089%-0.3% [1-4], and symptoms are frequently nonspecific (chest pain, dyspnea, cough, hypoxia, hemoptysis), making imaging central to diagnosis [1,2,5-8].

As early intervention can prevent irreversible ischemic injury, a high index of suspicion is warranted, particularly in patients with distorted lobar anatomy (e.g., giant bullae) whose imaging may mimic pneumothorax or atelectasis [2,3,6]. We present a case of partial right lower lobe torsion that was discovered during evaluation for an unrelated complaint.

This article was previously presented as a poster at the 2024 Southeastern Surgical Congress on February 3, 2024.

## Case presentation

**Figure 1:**
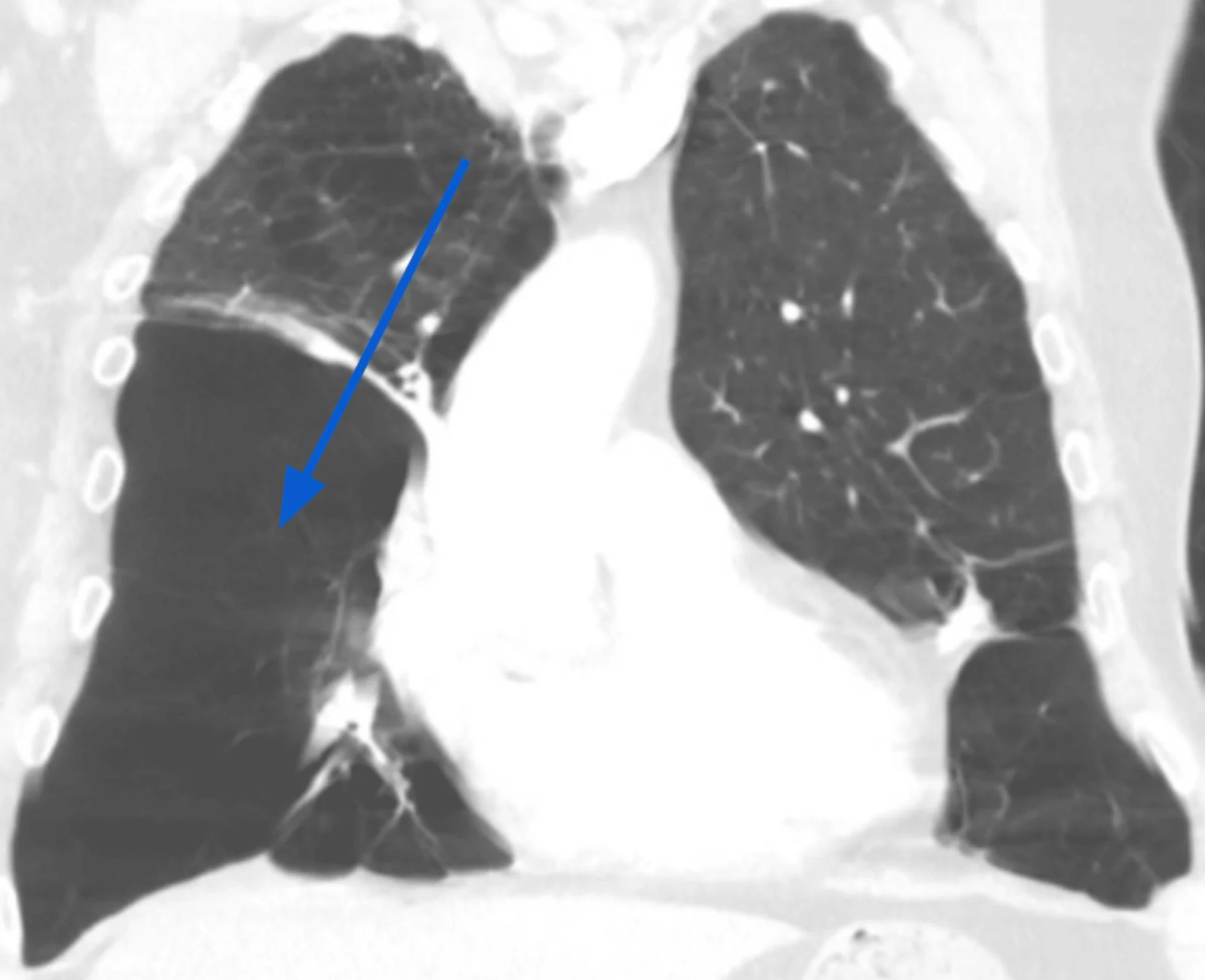
Coronal computed tomography obtained one month before surgery. The blue arrow identifies the large right-sided bulla.

A 68-year-old male with chronic obstructive pulmonary disease requiring 3 liters per minute of home oxygen presented to the emergency department for progressive bilateral foot swelling and erythema over one week associated with a new wound on the lateral aspect of the right foot. On examination, he had bilateral lower-extremity edema and a full-thickness ulceration of the lateral right foot. He denied chest pain and overt shortness of breath at the time of his presentation to the hospital. Past medical history was notable for a recent hospitalization for COVID-19 pneumonia one month earlier. During that admission, computed tomography (CT) reportedly demonstrated a large right-sided bulla (Figure 1), with thoracic surgery recommending outpatient follow-up after stabilization. He was a former cigarette smoker, although he denied recreational drug use.

On arrival, vital signs were unremarkable (blood pressure was 132/75 mmHg, heart rate was 95 beats per minute, respiratory rate was 18 breaths per minute, temperature was 36.7°C, and oxygen saturation was 94%). Examination revealed no respiratory distress and equal air entry bilaterally while on nasal cannula.

**Figure 2:**
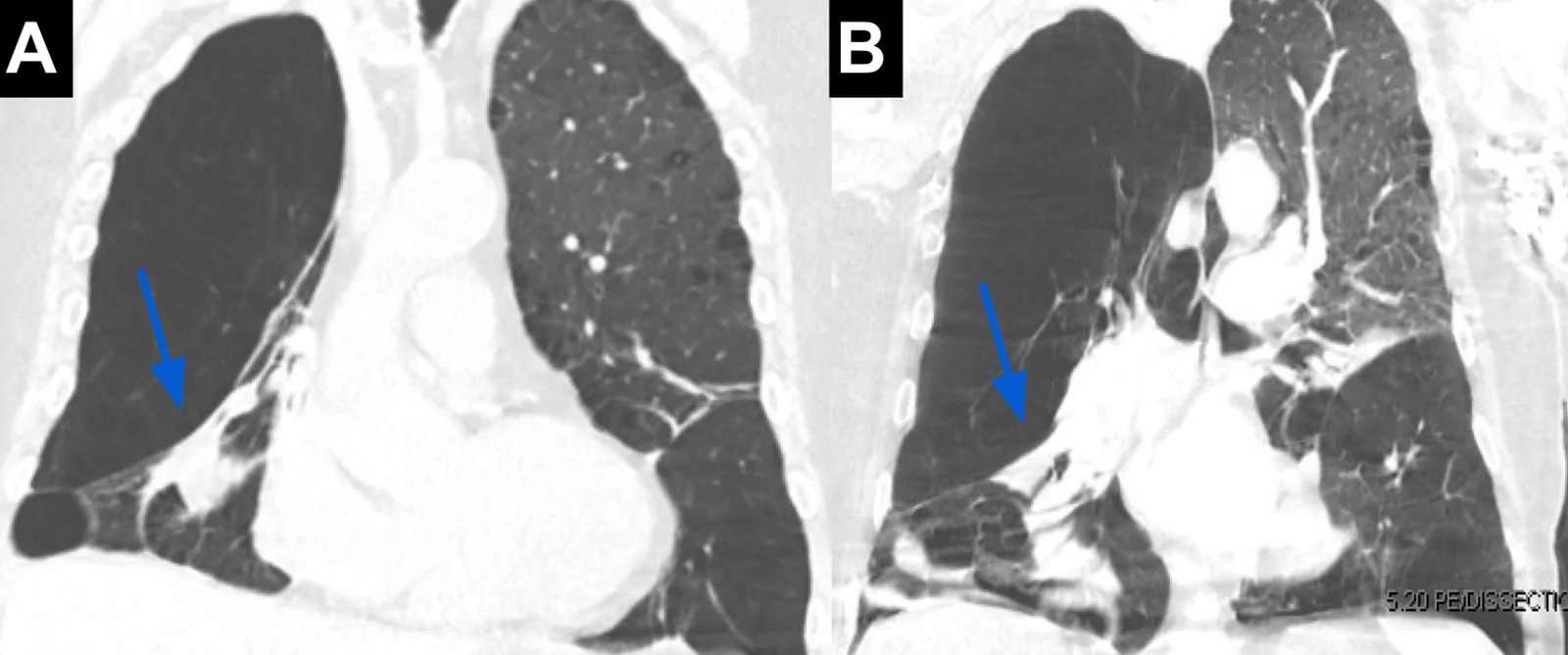
Coronal chest imaging demonstrating concern for torsion. (A) CT chest with intravenous contrast. The blue arrow indicates compressed and medially displaced right lower lobe parenchyma along the medial margin of the large bulla, which extends into the right upper lung region. (B) CT angiography. The blue arrow shows the abnormal orientation and swirling of the right hilar vessels.

Chest radiography raised concern for pneumothorax versus bulla, prompting thoracic surgery consultation. CT of the chest demonstrated an expanding right lower lobe bulla extending into the right upper lung zone, narrowing of the right bronchial tree, and suspected ball-valve obstruction without evidence of pneumothorax. As the distribution of the bulla had shifted compared with prior imaging, CT angiography was recommended to delineate the right pulmonary vessels, which demonstrated swirling of the vessels in the right hilar region (Figure 2).

After review of the CT findings, the patient was re-evaluated and consented for urgent bronchoscopy.

Operative and postoperative management

The patient initially underwent flexible bronchoscopy, which demonstrated compression of the right upper lobe bronchus. The right middle and lower lobe bronchi were also compressed and could not be traversed into their segmental branches, raising concern for torsion. The single-lumen endotracheal tube was exchanged for a double-lumen endotracheal tube, and positioning was confirmed with bronchoscopy. The patient was placed in the left lateral decubitus position; a right muscle-sparing thoracotomy was performed through the sixth intercostal space.

Upon exploration, there was partial twisting of the right lower lobe, after which the right lower and middle lobes were returned to their normal anatomic positions. A generous wedge resection of the right lower lobe was then performed to remove the bullous segment. Further resection was avoided because it would have left only the superior segment of the right lower lobe. Test inflation demonstrated satisfactory expansion of the right upper and middle lobes as well as the superior segment of the right lower lobe. A 28-French chest tube was placed, and the lung was expanded under direct visualization prior to closure. No intraoperative complications were appreciated.

Unfortunately, the postoperative course was complicated by a pleural effusion requiring decortication approximately two weeks later. The patient subsequently recovered well. At the final documented thoracic surgery follow-up two months after his operation, the patient was improving, although his supplemental oxygen requirement was 6 liters per minute. At his last clinical follow-up three years later, his oxygen requirement had returned to baseline, and he was referred to pulmonary rehabilitation.

## Discussion

Diagnostic challenges

The central feature of this case was the discordance between the patient’s nonthoracic/nonpulmonary presenting complaint, absence of acute respiratory symptoms, and markedly abnormal thoracic imaging. Dai et al. performed a pooled analysis of 109 patients with lung torsion, with dyspnea occurring in 38.4%, fever in 23.3%, and chest pain in 17.4%; the overall mortality was 8.3% [2].

Plain radiographic findings are often nonspecific and may include pulmonary opacity, volume loss, or an unexpected change in lobar position [3,9,10]. In the postoperative case series reported by Cable et al., five of seven patients had infiltrates and volume loss on chest radiography, whereas two had complete opacification [3]. The pooled analysis by Dai et al. identified worsening consolidation and abrupt truncation or tapering of a pulmonary artery as radiographic evidence of torsion [2]. Contrast-enhanced CT is therefore recommended when torsion is suspected because it can demonstrate abnormal lobar orientation, poor parenchymal enhancement, and tapering or interruption of the bronchovascular pedicle [10].

Predisposing anatomy and the pneumothorax mimic

Spontaneous lung torsion has been reported after spontaneous pneumothorax and after pleural effusion with associated atelectasis [11,12]. Giant bullous emphysema can closely resemble pneumothorax on chest radiography, and misinterpretation may actually result in unnecessary pleural drainage before CT clarifies the anatomy [13]. In the present case, the right lower lobe bulla expanded into the upper lung zone and was associated with marked bronchial narrowing and altered hilar vascular orientation. Although a direct causal relationship cannot be established, the imaging and operative findings suggest that the large bulla contributed to abnormal lobar displacement and partial torsion in this patient.

Role of computed tomography angiography and bronchoscopy

CT and bronchoscopy provide complementary evidence in suspected cases of lung torsion [2,6,10]. Bronchoscopy may reveal a narrowed lobar orifice, complete airway obstruction, extrinsic compression, or a tortuous bronchus [2]. However, nonspecific bronchoscopic findings do not exclude torsion. In a series by Taira et al., bronchoscopy showed no specific abnormality in four patients, whereas CT diagnosed torsion in all eight patients [6]. In this case, CT angiography demonstrated abnormal right hilar vascular orientation, while bronchoscopy showed severe compression of the right middle and lower lobe bronchi. The combined findings accelerated the decision to proceed with thoracotomy.

Operative management and tissue viability

Generally speaking, definitive management is surgical, with repositioning or detorsion considered when the involved lung remains viable, and resection favored when infarction or necrosis is present [2,6]. Dai et al. found similar survival after repositioning and direct resection, while emphasizing that viability should guide the operative approach [2]. Timely operative decision-making may preserve functional lung tissue in those with retained viability [6]. Conversely, delayed diagnosis has been associated with further pulmonary resection and prolonged hospitalization [3].

In this patient, it was deemed that the partially torsed lower lobe remained suitable for preservation. Detorsion successfully restored the normal position of the right lower and middle lobes, and limited wedge resection addressed the large bullous segment without sacrificing the remainder of the right lower lobe.

Strengths and limitations

This case report has several important limitations. First, as a single-patient case report, its findings may not be generalizable. Moreover, although the imaging and operative findings suggest that the large right lower lobe bulla contributed to abnormal lobar displacement and torsion, a direct causal relationship cannot be firmly established. Finally, intraoperative photographs are unavailable, limiting direct visual documentation of the torsion and subsequent detorsion. However, notwithstanding these shortcomings, the availability of serial cross-sectional imaging, bronchoscopic findings, operative confirmation, and long-term clinical follow-up strengthens the report and illustrates the diagnostic progression and management of this case.

## Conclusions

The present case demonstrates that lung torsion may be discovered during evaluation for an unrelated complaint. Discordance between the patient’s mild respiratory presentation and concerning thoracic imaging prompted further cross-sectional evaluation and operative exploration. Bronchoscopy and thoracotomy confirmed suspicion for lung torsion, and detorsion with limited wedge resection preserved viable lung tissue. Careful evaluation of atypical thoracic imaging in patients with abnormal pulmonary anatomy may expedite recognition and treatment.

## References

[1] Sahota RJ, Sankari A, Anjum F (2026). Lung torsion. StatPearls.

[2] Dai J, Xie D, Wang H, He W, Zhou Y, Hernández-Arenas LA, Jiang G (2016). Predictors of survival in lung torsion: a systematic review and pooled analysis. J Thorac Cardiovasc Surg.

[3] Cable DG, Deschamps C, Allen MS, Miller DL, Nichols FC, Trastek VF, Pairolero PC (2001). Lobar torsion after pulmonary resection: presentation and outcome. J Thorac Cardiovasc Surg.

[4] Hennink S, Wouters MW, Klomp HM, Baas P (2008). Necrotizing pneumonitis caused by postoperative pulmonary torsion. Interact Cardiovasc Thorac Surg.

[5] David A, Liberge R, Corne F, Frampas E (2016). Whole-lung torsion complicating double lung transplantation: CT features. Diagn Interv Imaging.

[6] Taira N, Kawasaki H, Takahara S (2018). Postoperative lung torsion with retained viability: the presentation and surgical indications. Heart Lung Circ.

[7] Duan L, Chen X, Jiang G (2012). Lobar torsion after video-assisted thoracoscopic lobectomy: 2 case reports. Thorac Cardiovasc Surg.

[8] Kita Y, Go T, Nii K, Matsuura N, Yokomise H (2017). Spontaneous torsion of the right upper lung lobe: a case report. Surg Case Rep.

[9] Felson B (1987). Lung torsion: radiographic findings in nine cases. Radiology.

[10] Childs L, Ellis S, Francies O (2017). Pulmonary lobar torsion: a rare complication following pulmonary resection, but one not to miss. BJR Case Rep.

[11] Irie M, Okumura N, Nakano J (2014). Spontaneous whole-lung torsion after massive pleural effusion and atelectasis. Ann Thorac Surg.

[12] Berkmen YM (1985). Uncomplicated torsion of the right upper lobe secondary to spontaneous pneumothorax. Chest.

[13] Samanta RP, Agarwal S, Sengupta S (2022). Giant bullous emphysema mimicking spontaneous pneumothorax. Cureus.

